# Ultrasound-triggered release of an active Wnt signalling agent (6-bromoindirubin-3-oxime) from perfluorocarbon nanodroplets

**DOI:** 10.1039/d5pm00186b

**Published:** 2026-07-13

**Authors:** Anastasia Polydorou, Qiang Wu, Aya Ben Issa, Jonathan P. May, Sara Ferri, Dario Carugo, Eleanor Stride, Nicholas D. Evans

**Affiliations:** a Centre for Human Development, Stem Cells and Regenerative Medicine, Bone and Joint Research group, Faculty of Medicine, University of Southampton Southampton UK n.d.evans@soton.ac.uk; b Bioengineering Sciences Group, Institute for Life Sciences, Faculty of Engineering and Physical Sciences, University of Southampton Southampton UK; c Institute of Biomedical Engineering, University of Oxford Oxford UK eleanor.stride@eng.ox.ac.uk; d Nuffield Department of Orthopaedics, Rheumatology and Musculoskeletal Sciences (NDORMS), Medical Sciences Division, University of Oxford Oxford UK

## Abstract

Ultrasound-activated perfluorocarbon nanodroplets (NDs) have potential as circulating agents for non-invasive, targeted drug delivery. NDs can be induced to cavitate under focussed ultrasound stimulation, releasing a therapeutic payload. They have been explored widely as a means of drug delivery in oncology but there is less information available on their use in regenerative medicine. Here, we tested the hypothesis that NDs could be fabricated to incorporate an agonist of Wnt signalling, a pathway common to many regenerative tissue-specific processes, and to release it in active form upon ultrasound stimulation. NDs were formed by ultrasonic emulsification of perfluorobutane in the presence of 6-bromoindirubin-3-oxime (BIO) and phospholipids: DSPC and DSPE-PEG2000. The nanodroplet formulation (∼200 nm diameter and 5 × 10^9^ mL^−1^) was stable in aqueous suspension for up to 10 days at 4 °C, but gradual passive release of BIO was observed over a period of hours (15–30% release in 24 hours) in serum-containing growth medium at 37 °C, as measured by high pressure liquid chromatography. NDs were not toxic to either primary or continuous bone-derived cells and induced signalling in a 3T3 Wnt reporter cell line, indicating that the released BIO was active. Rapid release of BIO was achieved by exposing NDs to ultrasound. 25% BIO release was observed after 30 s of exposure to ultrasound (0.9 MHz centre frequency, 0.75 MPa peak negative pressure, 1 kHz pulse repetition frequency, 10% duty cycle) and activated Wnt signalling in cells. Our data indicate that NDs may be used to incorporate and deliver a Wnt pathway activator in response to ultrasound stimulation and that they have potential as drug delivery agents for regenerative medicine applications.

## Abstract 2

Ultrasound-triggered vaporisation of BIO-loaded perfluorobutane nanodroplets enables rapid release of active BIO, enhancing Wnt/TCF–LEF reporter signalling in cells and supporting spatially controlled delivery for regenerative medicine.

## Graphical abstract

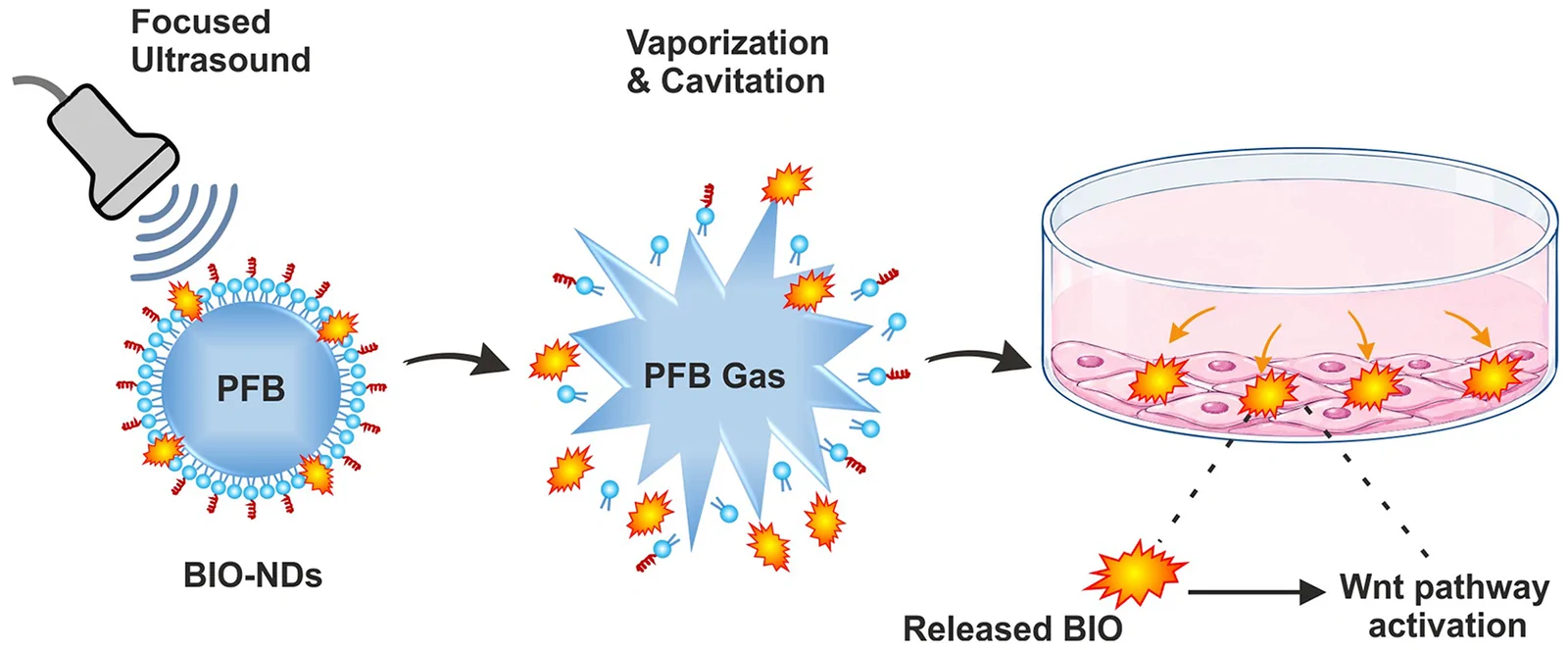

## Introduction

Perfluorocarbon nanodroplets (PFC-NDs) have potential as circulating agents for non-invasive, targeted drug delivery in applications including oncology and regenerative medicine. PFC-NDs can be made by emulsification and stabilisation of volatile fluorocarbons such as perfluoropentane in aqueous solutions using amphiphilic molecules such as phospholipids.^1^ The liquid perfluorocarbon in these sub-micrometre diameter droplets is stabilised in a super-heated state and, upon exposure to ultrasound pressure waves, can be induced to vaporise to form gaseous microbubbles.^2–4^ Because PFC-NDs can be modified to incorporate biologically active compounds, ultrasound can be used to trigger local drug release in the region where the PFC-NDs are present. In addition, the energetic vaporisation of PFC-NDs and the subsequent microbubble expansion and contraction in an ultrasound field lead to enhanced drug convection and tissue penetration.^5^

PFC-NDs offer several advantages over microbubbles: being smaller, they can have much greater stability in circulation (hours compared to minutes for microbubbles of similar composition),^6^ they can readily perfuse the smallest capillaries, and it is believed that in tissues with disorganised or developing vasculature (such as in tumours or in inflamed tissue) they can passively transit the endothelium and become concentrated in the extravascular space.^7^ PFC-NDs are also taken up by a wide range of cell types, suggesting that intracellular targeting may also be possible.^8–10^

The majority of published reports utilising PFC-NDs focus on delivery of chemotherapeutic agents for cancer therapy. In an early study, doxorubicin was incorporated in di-block copolymer micelles containing perfluoropentane.^1^ Upon heating or ultrasound stimulation, doxorubicin was released, leading to enhanced cell uptake *in vitro* and reduction of tumour volume *in vivo*. Variations and refinements to this approach have been developed over subsequent years, with novel strategies including co-delivery of other several therapeutic agents^11^ and modification of the PFC-ND with motifs that recognise and bind to tumour specific receptors.^4^ In addition, PFCs are known to have a very high solubility for gases, including oxygen, which has led to their use over the years as blood substitutes.^12^ This property has been exploited to co-deliver oxygen to hypoxic tumours, in order to sensitise otherwise hypoxic tumour environments to chemotherapeutic drugs dependent on oxygen for their toxicity.^13^

Despite this progress, there are relatively few studies that have explored the potential of PFC-NDs in other applications. Examples include in delivering localised anaesthesia to the brain,^14^ the treatment of degenerative disc disease,^15^ and neuromodulation.^16^ Recently, Shar *et al.* developed a targeted approach for treating osteoporosis by using human serum albumin to emulsify perfluorohexane in the presence of siRNA for cathepsin K and alendronate – two antiresorptive drugs – and observed inhibitory effects on osteoclasts.^17^ It is likely that PFC-NDs may have significant potential in a range of clinical applications where non-invasive, site-specific delivery is key.

We are exploring the potential use of PFC-NDs for site-specific drug delivery in regenerative medicine, including in bone repair. In these situations, local activation of signalling pathways may be particularly advantageous. For example, in bone repair, localised activation of bone morphogenetic protein (BMP)^18^ or Wnt signalling^19^ pathways promotes bone formation. However, this relies on implantation or injection of materials containing growth factors, which is invasive, leads to physical disruption or modification of the local environment, and therefore may not be conducive to repeated administrations. Although there have been limited studies of ultrasound-activated agents in regenerative medicine, Crasto *et al.* showed that circulating BMP2-containing liposomes could be activated locally with remote ultrasound and promote local bone formation in rats.^20^ Moreover, Bez *et al.* showed that locally implanted microbubbles integrated within a collagen scaffold promoted local ultrasound-mediated transfection of the gene for BMP2 and enhanced bone formation in minipigs.^21^ Wnt proteins or other molecules that activate Wnt signalling have yet to be explored, but this pathway is a potent inducer of bone differentiation that is dependent on temporally defined activation.^19,22,23^

In an effort to develop PFC-ND emulsions suitable for local activation of Wnt signalling in regenerative medicine applications, such as in bone repair, in this study we fabricated perfluorobutane NDs, incorporated a Wnt agonist in them, and tested the hypothesis that ultrasound could be used to activate release and induce Wnt signalling in a cell culture model.

## Experimental

### Materials

The following compounds were purchased from Sigma-Aldrich Inc.: 1,2-distearoyl-*sn-glycero*-3-phosphocholine (DSPC), 1,2-distearoyl-*sn-glycero*-3-phosphoethanolamine-*N*-[methoxy(polyethylene glycol)-2000] (DSPE-PEG2000), (2′*Z*,3′*E*)-6-bromoindirubin-3′-oxime (BIO), phosphate-buffered saline (PBS), propylene glycol, glycerol, ethanol, methanol, trifluoroacetic acid, acetonitrile, and 3,3′-dioctadecyloxacarbocyanine perchlorate (DiO). These chemicals were used without further purification. Perfluorobutane (PFB, FluoroMed, L.P. USA) was also used without further purification. Ultrapure deionized water was obtained from a Millipore Milli-Q plus system (Millipore S.A.S., France).

### Formulation of BIO-loaded perfluorobutane NDs

Initially, a 0.8 mL solution containing 20 mg of 90 mol% DSPC and 10 mol% DSPE-PEG2000 dissolved in chloroform (yielding a total lipid concentration of 25 mg mL^−1^) was prepared, followed by evaporation to form a thin dry film. The generation of BIO-loaded PFB NDs involved the addition of BIO to the lipidic chloroform solution before evaporation, to achieve a final molar concentration of 1.4 mM. The labelled BIO-NDs were formed by adding DiO to the lipidic chloroform solution to achieve a final molar concentration of 10 μM.

**Fig. 1:**
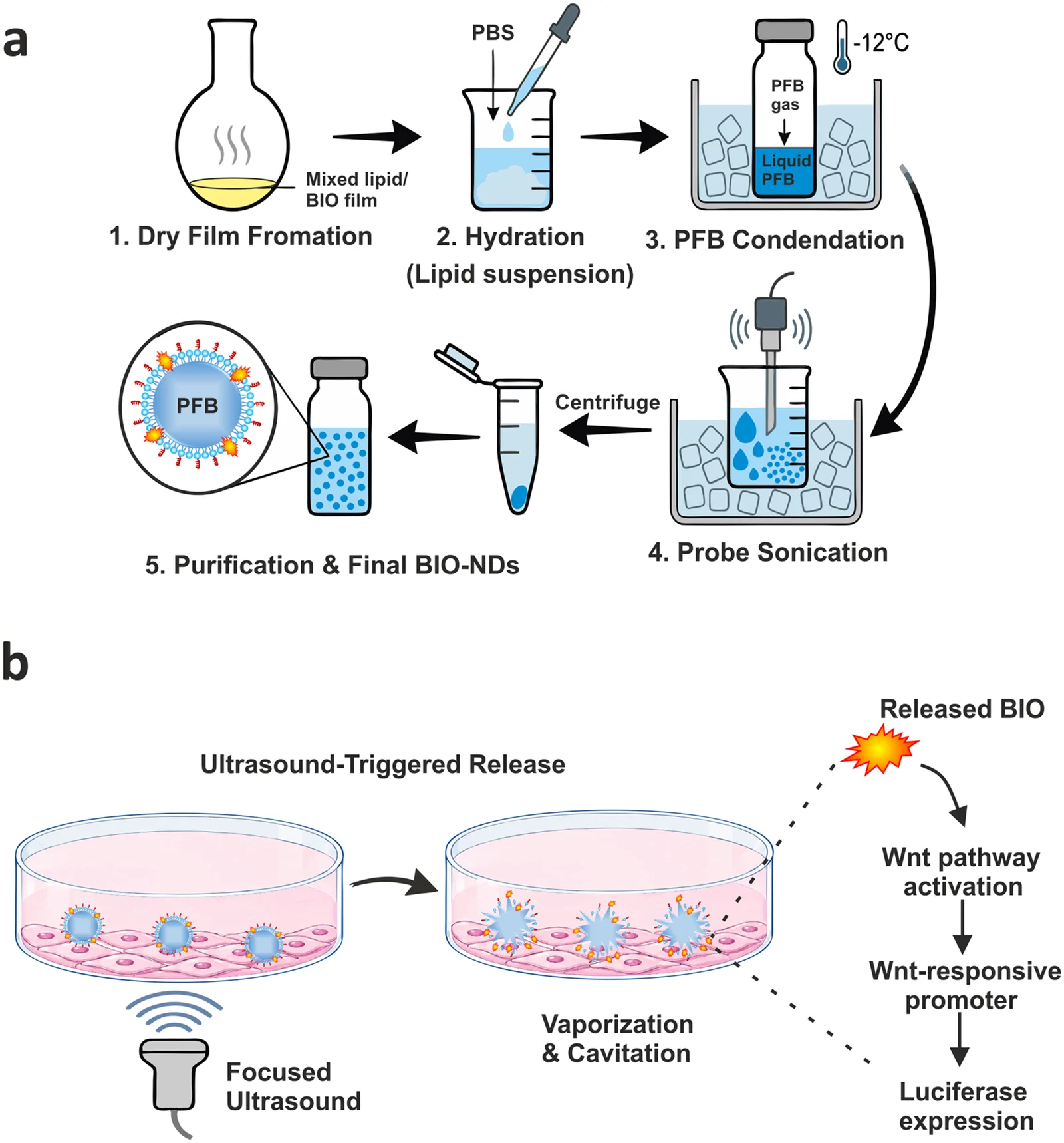
A schematic diagram showing the method of production of BIO-nanodroplets by dry lipid/BIO film production, hydration and PFB condensation prior to sonication (a) and their use in this study as ultrasound-activated release depots for active BIO in cell culture assays (b).

Subsequently, the resulting lipid films were hydrated using 4 mL of a solution comprising PBS/propylene glycol/glycerol in a volume ratio of 16 : 3 : 1. The production of the NDs commenced with the condensation of PFB by introducing PFB gas into a scintillation vial, which was cooled to −12 °C in an ethanol/ice bath. Subsequently, 100 µL of the resulting PFB liquid was incorporated into the lipid suspension. The mixture underwent sonication using a probe sonicator (Q125, QSonica, LLC., USA; tip diameter: 3 mm) within an ethanol ice bath maintained at temperatures ranging from −7 °C to −12 °C, operating at 50% amplitude for 3 minutes (125 W, 20 kHz, pulsed mode: 2 s on and 4 s off). To eliminate excess free lipids, the resulting ND emulsion was centrifuged at 10 000 rpm (11 292*g*) for 6 minutes, followed by resuspension in fresh PBS. This centrifugation and washing process was repeated three times. Finally, the prepared NDs were stored at 4 °C for future use. A schematic diagram showing the methods of production and usage is shown in Fig. 1.

### Characterization of BIO-loaded perfluorobutane nanodroplets (BIO-NDs)

The morphology of the BIO-NDs was characterized *via* cryogenic transmission electron microscopy (Cryo-TEM) utilizing a Talos Arctica TEM machine (Thermo Fisher Scientific, Hillsboro, Oregon; USA). For imaging, the BIO-ND sample was applied onto Lacey carbon grids and subsequently frozen using a Thermo Fisher Vitrobot Mark IV. The nanodroplet size from TEM images was measured employing ImageJ software (National Institutes of Health, Bethesda, MD, USA).

The concentration and size of BIO-NDs dispersed in PBS were quantified utilizing nanoparticle tracking analysis (NTA) conducted on a NanoSight LM-20 instrument (Malvern Panalytical, Worcestershire, UK) with NTA software (Version 3.0, Build 0066, Malvern Instruments). Three independent samples were analysed for each of the three BIO-ND batches. The hydrodynamic diameter of BIO-NDs was also quantified utilizing dynamic light scattering (DLS) with a Zetasizer Nano ZS instrument (Malvern Panalytical, Worcestershire, UK) at a temperature of 20 °C. Each batch was subjected to analysis using three independent samples. The stability of BIO-NDs was assessed in PBS at two different temperatures: storage temperature (4 °C) and physiological temperature (37 °C). Changes in particle size and concentration were determined over periods of up to 10 days at 4 °C and 24 hours at 37 °C, using NTA, in PBS.

The amount of BIO encapsulated in BIO-NDs was measured using a high-pressure liquid chromatography (HPLC) system. 400 µL of ethanol were added to 100 µL of dispersed BIO-NDs (1 : 100 dilution in PBS). HPLC analyses were performed on an Agilent 1260 Infinity II (Agilent, California, USA) with UV detection at 500 nm. A ZORBAX RRHD StableBond C18 column (2.1 mm × 100 mm, 1.8 µm; Agilent, California, USA) was used for chromatographic separation. The mobile phase was composed of 0.1% (v/v) trifluoroacetic acid in water (A) and 0.1% (v/v) trifluoroacetic acid in acetonitrile (B). Gradient elution was performed at a flow rate of 0.3 mL min^−1^ and a temperature of 25 °C, with a linear gradient of 20%–100% of B over 5 minutes. 5 µL of sample were injected for each run.

The quantity of BIO was then determined by comparing the light absorption with a previously constructed standard curve. This curve was generated by measuring the absorption of BIO at 500 nm in a solution consisting of PBS and ethanol at a ratio of 1 : 4, across various concentrations of BIO. Encapsulation efficiency (EE, %) was calculated by the following eqn (1):1
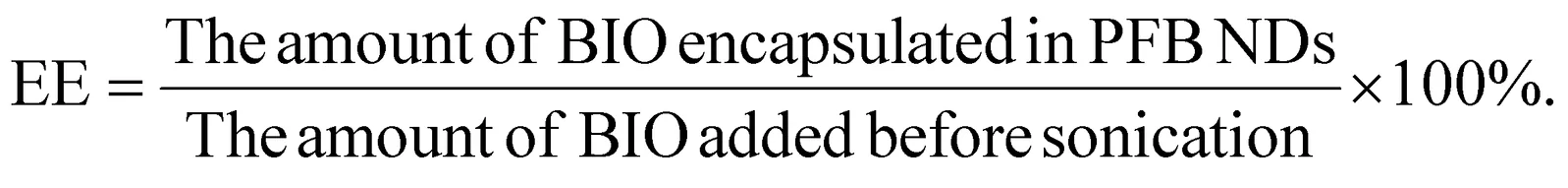


### Analysis of ultrasound-stimulated release of BIO from NDs

To assess release of BIO from BIO-NDs induced by ultrasound stimulation, BIO-NDs were diluted in Dulbecco's modified Eagle's medium (DMEM) (Lonza, UK) to a final concentration of 20 µM BIO (corresponding to approximately 2 × 10^9^ NDs mL^−1^), or control NDs (at the same concentration) were prepared. The ND samples were stimulated by ultrasound at 1 MHz, 1.1 MPa (peak negative pressure), 5000 cycles, 10% duty cycle, for durations of 15, 30, 60, 90, 120, 180, or 300 seconds, using an *in vitro* “SAT2” system as reported previously.^24^ In this system, a 1 MHz centre frequency ultrasound source (Imasonic 8233A101, 40 mm diameter, 120 mm radius of curvature) is located at the base of a cylindrical water tank, maintained at 37 °C for these experiments, and driven by a radio frequency amplifier (1020L, E&I Ltd, 200 W power amplifier) connected to a waveform generator (33250, Agilent). The SAT2 cell exposure compartment is established through the aseptic assembly of a deformable polydimethylsiloxane (PDMS) lid onto a designated culture dish (35 mm in diameter, Ibidi, Germany). The acoustic signal was detected by a single element focussed transducer used as a passive cavitation detector (5 MHz, V326-SU, Olympus Panametrics, Southend-on-Sea, Essex, UK) aligned with the centre of the cell culture dish. Detected signals were passed through a 2 MHz high-pass filter (Allen Avionics, River Grove, IL, USA) to remove the fundamental 1 MHz frequency and were recorded *via* an oscilloscope (Handyscope HS3, TiePie Engineering, Sneek, Netherlands). After ultrasound exposure, 150 μL samples were extracted from the dish and transferred into 1.5 mL Eppendorf™ tubes. Samples were then centrifuged at 10 000*g* to remove unvaporised NDs, and the amount of BIO in the supernatant was measured using HPLC as reported above. For experiments examining the effect of the BIO released from ultrasound-activated BIO-NDs on cellular Wnt signalling activity, BIO-NDs or control NDs were exposed to ultrasound for 300 s as above, the supernatant was collected, stored at 4 °C, and subsequently added to Wnt reporter assay medium at dilutions of 1, 0.5, 0.25 and 0.1 (corresponding to initial ND concentrations of 2 × 10^9^, 1 × 10^9^, 5 × 10^8^, and 2 × 10^8^ mL^−1^ respectively) as reported in the ‘Wnt pathway activity assay’ section below.

### Cell culture

MG63 cells were cultured in DMEM containing l-glutamine and 4.5 g L^−1^ glucose supplemented with 10% (v/v) fetal bovine serum (FBS) (Gibco, ThermoFisher, UK) and 1% (v/v) penicillin–streptomycin containing 10 000 units per mL of penicillin and 10 000 units per mL of streptomycin (Lonza, UK). Primary human bone marrow stromal cells (HBMSCs) were isolated from bone marrow samples obtained from adult patients undergoing hip-replacement surgery at Southampton General Hospital and Spire Southampton Hospital.^25^ All work involving human-derived tissue/cells was performed in accordance with the Declaration of Helsinki, the UK Human Tissue Act 2004, the UK Policy Framework for Health and Social Care Research, and relevant institutional governance and data-protection requirements. Ethical approval for the collection and use of human tissue was granted the UK National Ethics Committee (REC reference: 18/NW/0231), with local governance approval from the University of Southampton Ethics and Research Governance Office (ERGO reference: 31875.A12; project title: “Skeletal Tissue Engineering”). Written informed consent was obtained from all participating patients before tissue donation. All samples were handled and processed in accordance with the approved protocols. Cells that had been passaged fewer than six times were cultured in minimum essential medium Eagle – alpha modification (α-MEM) (Lonza, UK) containing l-glutamine and 4.5 g L^−1^ glucose supplemented with 10% FBS (v/v) (Gibco, ThermoFisher, UK) and 1% (v/v) penicillin–streptomycin. BIO activity was measured using a commercially-sourced 3T3 cell line (Enzo Life Sciences, New York, USA). This line is genetically engineered with a firefly luciferase enzyme promoter region under the control of Wnt expression.^26^ 3T3 cells were cultured in DMEM supplemented with 5% (v/v) FBS, 0.5% (v/v) P/S, and 20 mM HEPES buffer. All cell lines were passaged at 37 °C, 5% CO_2_ and 95% humidity and sub-cultivated at ∼80% confluency.

### Cell metabolism, viability and uptake assays

MG63 or BMSCs cells were seeded in 96 well plates with dark-walls and clear bottoms (Corning, Thermo Fisher Scientific, UK) at a seeding density of 60 000 cells per cm^2^. Cells were allowed to attach and grow for 24 hours prior to experiments. They were then incubated with ND dispersions at concentrations of up to 1 × 10^9^ NDs mL^−1^ for either 20 minutes, 40 minutes, or 24 hours. At each timepoint, the media was aspirated and cells were washed twice with pre-warmed PBS. 100 μL of 10% (v/v) alamarBlue™ reagent diluted in basal media, was added to each well. Blank wells (no cells or reagent) were included, which served as the background level to later normalise the readings. Cells were incubated with alamarBlue™ for between 2 and 3.5 hours to allow the alamarBlue™ fluorescence to develop. alamarBlue™ gives a measure of cell viability as its active component, resazurin, is non-toxic and diffuses through cell membranes. Live, metabolically active cells reduce resazurin to the fluorescent active compound, resorufin, therefore providing a measure of cell viability *via* MA that was recorded as fluorescence intensity. Fluorescence was detected by a GloMax® Discover (Promega, UK) plate reader with fluorescence intensity read at 520 nm excitation and between 580 nm and 640 nm emission wavelengths. For direct assessment of viability, after 24 hours incubation with NDs, cells were then washed with PBS and stained with a solution containing Cell Tracker™ Green (10 µg mL^−1^) and Ethidium Homodimer-1 (5 µg mL^−1^) (Thermo Fisher Scientific) for 2 hours. Cells were subsequently washed with PBS and imaged using an EVOS fluorescence microscope. Live (CellTracker Green–positive; Ex 492 nm, Em: 518 nm) and dead (Ethidium Homodimer-1–positive; Ex: 568 nm, Em: 580–620 nm) cells were counted, and cell viability was calculated as the percentage of live cells relative to the total number of cells. To test nanodroplet association with cells, fluorescent nanodroplets were made as described above. MG63 cells were seeded at a density of 60 000 cells per cm^2^ in Ibidi dishes with a polymer coverslip bottom (35 mm in diameter and 12 mm high; Ibidi, Martinsried, Germany). After 24 hours of culture, cells were incubated with ND dispersions at concentrations of up to 1 × 10^9^ NDs mL^−1^ for an additional 24 hours. Cells were subsequently washed with PBS and stained with CellTrackerTM Deep Red (1 µM) for 1 hour, followed by Hoechst (1 µM) for 10 minutes. Images were acquired using a Leica TCS SP5 confocal microscope. *Z*-Stack images were acquired at 0.5 µm intervals using a 63× objective.

### Wnt pathway activity assay

3T3 cells, maintained as described above, were seeded at 15 000 cells per well in opaque 96-well plates and cultivated for 24 hours in growth medium. For activity assays, formulations of NDs at concentrations up to 1 × 10^9^ NDs mL^−1^, BIO at concentrations up to 10 µM, supernatant of medium incubated with NDs, or supernatant of either US-activated or non-US-activated NDs, were added to the wells in DMEM for 18 hours. Lysis/luciferase substrate was added to each well at 1 : 1 dilution for 5 min at room temperature before luminescence was measured using a GloMax® Discover (Promega) at 580–620 nm emission. To normalise to cell numbers, the cell lysates were analysed for dsDNA content using Quant-iT™ PicoGreen® dsDNA reagent (Thermo Fisher Scientific, UK), following the manufacturer's recommendations.

### US stimulation of cells directly in the presence of BIO-NDs

For ultrasound stimulation experiments, 3T3 cells were cultured in 35 mm ibidi® dishes (IB-81156, μ-Dish 35 mm, high ibiTreat, Thistle Scientific, UK). Prior to cell seeding, dishes were coated with fibronectin by addition of 1 mL of a 50 μg mL^−1^ fibronectin solution (in PBS), for 2 hours at 4 °C followed by aspiration. 3T3 cells were seeded at 60 000 cm^−2^ and cultured for 24 hours prior to use. For ultrasound stimulation, the plastic lid of the plate was replaced with a custom-designed deformable PDMS lid following a previously described protocol.^27^ The lid has two 1.2 mm diameter apertures, positioned to facilitate priming of the dish using an 18-G blunt needle connected to a syringe. This also enables removal of air pockets from the dish. Medium containing BIO or BIO-NDs at concentrations stated in the Results section were added. The dish was then inserted within a custom designed instrument^24^ (SAT2 system), where it was exposed to ultrasound at a frequency of 0.9 MHz, 0.75 MPa (peak negative pressure), 1000 Hz PRF, 10% duty cycle, for 30 seconds using a H151 transducer (Sonic Concepts, Washington, USA) focussed to the surface of the cell culture plate. The SAT2 chamber was fitted with a top lid that contained a hole for insertion of an immersion transducer (5 MHz, V326-SU, Olympus Panametrics, Southend-on-Sea, Essex, UK) to perform passive cavitation detection (PCD). The acoustic data recorded by this transducer were processed in MATLAB (The MathWorks Inc., Natick, MA, USA) to determine the change in amplitude and frequency content of the acoustic emissions over time, and hence of ND's behaviour upon exposure to ultrasound.

The ibidi® dish was then removed from the SAT2, excess medium was removed to leave 2 mL of medium in the dish, and the dish was incubated for ∼18 hours prior to analysis of Wnt pathway activation by luciferase activity, as described above. In parallel, BIO released in the medium was quantified using HPLC. For this purpose, a 150 μL sample of the medium was extracted from the cell culture dish and transferred into a 1.5 mL Eppendorf™ tube. Subsequently, 600 µL of methanol was added to the supernatant, and the solution was gently shaken. After centrifugation, the supernatant was transferred into a new 1.5 mL Eppendorf™ tube. The liquid was then evaporated using a centrifuge concentrator (Concentrator plus, Eppendorf, UK) overnight at 30 °C. Following evaporation, 300 μL of a mixture of ethanol and PBS, at a ratio of 4 : 1, was added to the resulting dried precipitate. The mixture was then analyzed by HPLC. The amount of BIO released in the medium was calculated by comparing the results with a previously determined standard curve.

### Analysis of passive release of BIO from NDs

ND stability may be affected by the presence of serum components in cell growth medium. To determine the rate of passive release of BIO, BIO-NDs at a concentration of 1 × 10^11^ mL^−1^ (corresponding to a bulk concentration of 1 mM BIO) were diluted at (v/v) 1 : 50, 1 : 100 and 1 : 200 in either growth medium or PBS and incubated at 37 °C for 0, 1, 4, 24 and 48 hours. At each time point, 1 mL was removed and centrifuged at 10 000*g* for 5 min to remove the NDs, and the supernatant was removed and stored at 4 °C. The amount of BIO in the supernatant was then measured by HPLC or its activity assessed by direct incubation of supernatant samples with 3T3 cells, as reported above.

### Statistical analysis

Comparisons between experimental groups were made using either one-way or two-way-ANOVA with a *post-hoc* Tukey test following testing for normality using a Shapiro–Wilk test. Unpaired Student-*t*-tests were used when comparing the means between two unpaired variables. All statistical significance was accepted at a *p* value of 0.05.

## Results

### Formulation and characterisation of perfluorobutane NDs

**Fig. 2:**
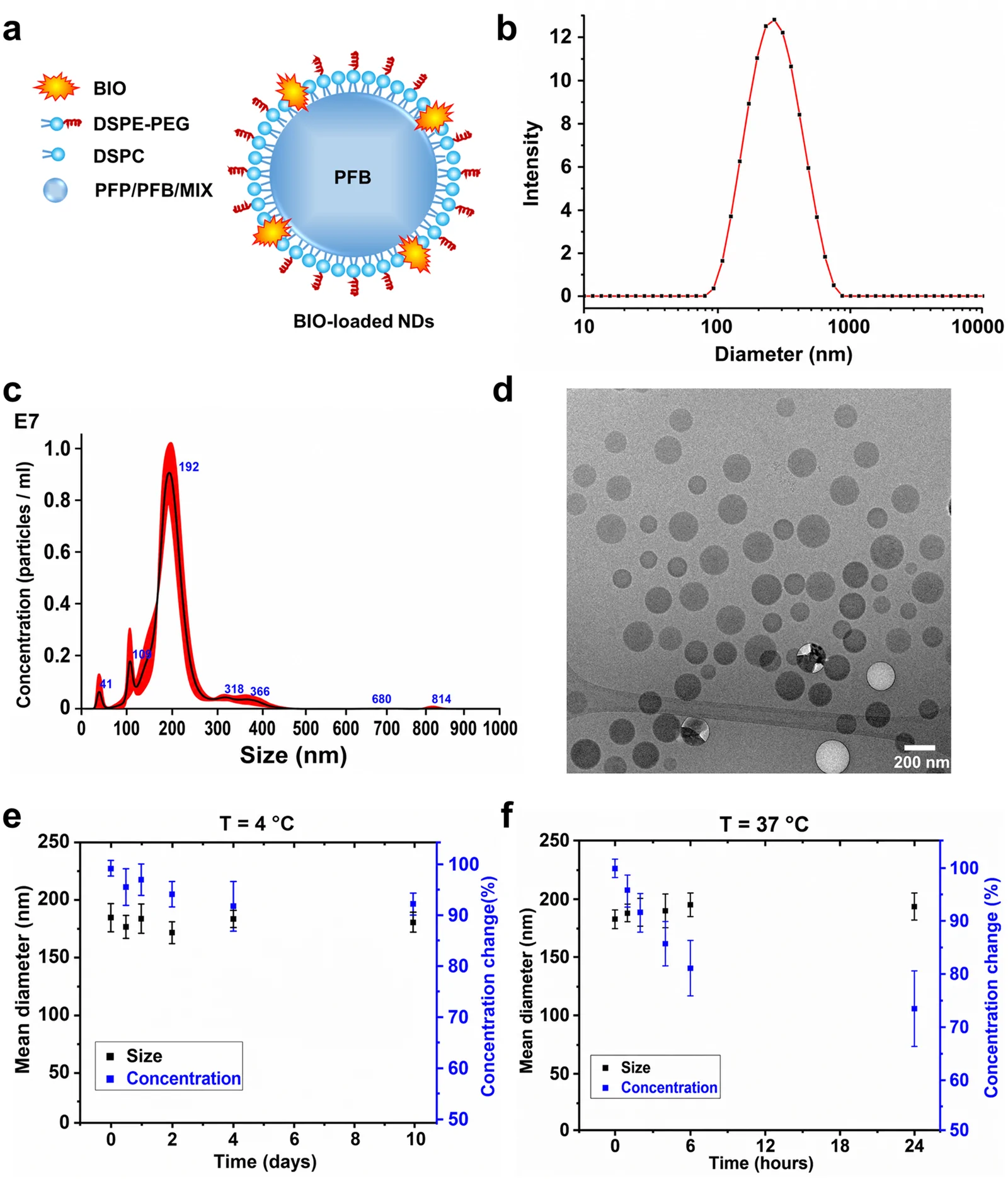
Characterization of BIO-NDs. (a) Schematic of a BIO-ND; the size distribution of PFB NDs measured by (b) DLS and (c) Nanosight, where the red shading indicates measurement variability across 3 runs and blue figures indicate local peak values; (d) cryo-TEM image of PFB NDs. Scale bar is 200 nm. (e) The ND size and concentration change over time at 4 °C and (f) the ND size and concentration change over time at 37 °C, measured by NTA. Data points are mean values of 3 separate measurements, with error bars representing the corresponding standard deviation.

We recently reported on the incorporation and acoustically-activated release of a fluorophore from ND emulsions of PFB and perfluoropropane (PFP),^28^ suggesting the potential of perfluorocarbon NDs for ultrasound-mediated drug delivery. In this study we attempted encapsulation of a small-molecule agonist of the Wnt signalling pathway, BIO. Activation of Wnt signalling plays a key role in the regeneration of many tissues, including bone. BIO-loaded NDs (Fig. 2a) had a mean diameter of 225.1 ± 15.2 nm measured by DLS (Fig. 2b) and 182.4 ± 13.8 nm measured by NTA (Fig. 2c), which was not significantly different from unloaded NDs (215.1 ± 10.9 nm and 175.7 ± 18.5 nm, respectively). Under cryo-TEM imaging, circular electron-dense objects were predominantly visible with diameters ranging from 50 to 300 nm, likely reflecting PFB-containing NDs. However, some of these objects were not electron-dense, appearing light in colour, which may reflect the presence of air bubbles and/or vaporised NDs (Fig. 2d).

To determine stability in storage conditions, BIO-NDs were stored at 4 °C for 10 days in PBS buffer and their size and concentration measured by NTA. The initial concentration of BIO-NDs was measured at 2.0 ± 0.3 × 10^10^ NDs mL^−1^. There was a small decrease in concentration over time in these conditions but no significant increase in mean ND diameter (Fig. 2e). To determine stability at physiological temperature, the BIO-NDs were stored at 37 °C for 24 hours, again in PBS. The initial concentration of BIO-NDs was measured at 2.0 ± 0.3 × 10^10^ NDs mL^−1^. This led to a significant decline in concentration (a decrease of 26.5% at 24 hours compared to after fabrication) and to a small and not significant increase in mean diameter from 183.2 ± 8.1 nm to 194.1 ± 11.6 nm (*p* = 0.253; *n* = 3), as shown in Fig. 2f. No microbubble formation was observed at 4 °C. At 37 °C, a small number of microbubbles did form indicating some spontaneous vaporisation. These bubbles were removed by centrifugation before the NTA measurements were performed, to avoid biasing the size measurements.

**Fig. 3:**
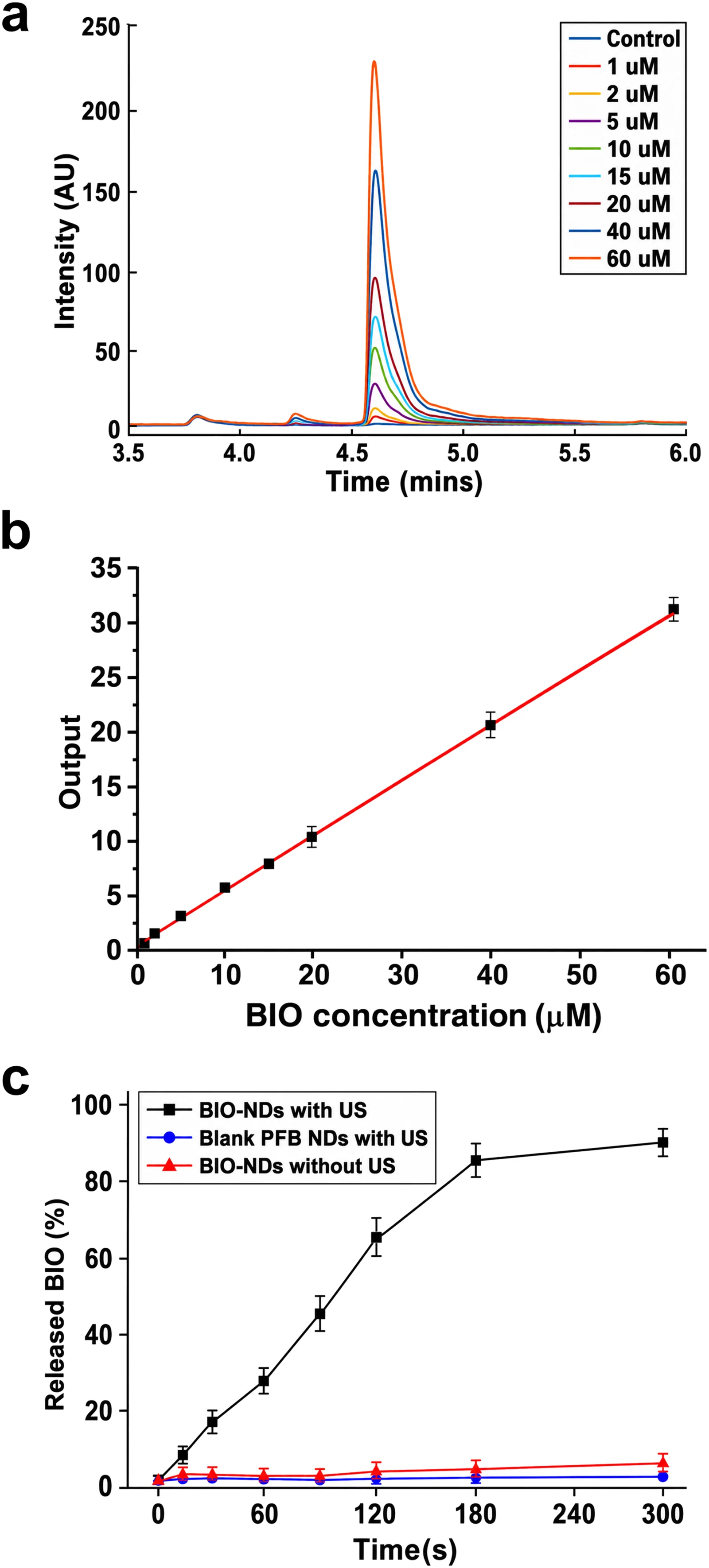
BIO-NDs can be induced to release BIO under ultrasound stimulation. (a) HPLC trace shows a BIO-specific peak between 4.5–5.5 min; (b) standard curve showing the fluorescence intensity *vs.* BIO concentration relationship; (c) release profiles of BIO from BIO-NDs and blank PFB NDs with varying ultrasound exposure times, and from BIO-NDs without ultrasound exposure. Data points are mean values of 3 separate measurements, with error bars representing the corresponding standard deviation. The frequency, peak negative pressure, number of cycles, and duty cycle of ultrasound were set to 1 MHz, 1.1 MPa, 5000 cycles and 10%, respectively.

The encapsulation efficiency of BIO in PFB NDs was determined to be 85.6 ± 3.5%. HPLC was used to determine the amount of BIO released from BIO-NDs following ultrasound exposure. The HPLC trace shows a peak corresponding to BIO, located between 4.5–5.5 min (Fig. 3a). There was a strongly linear relationship (*R*^2^ = 0.999) between BIO concentration and HPLC output. Exposure to ultrasound (1 MHz, 5000 cycles, and 10% duty cycle) with a 1.1 MPa peak negative acoustic pressure produced a time dependent release of BIO from NDs, which was not seen in control samples without drug and/or that were not exposed to ultrasound (Fig. 3c). After 1 min of ultrasound exposure, 27.5% of the encapsulated BIO was released from the NDs, with 66.2% and 89.1% released within 2 min and 5 min, respectively. These data indicate that NDs are stable in aqueous buffer and can be induced to release a drug payload under ultrasound stimulation.

### NDs do not induce cytotoxicity in bone-derived cells

**Fig. 4:**
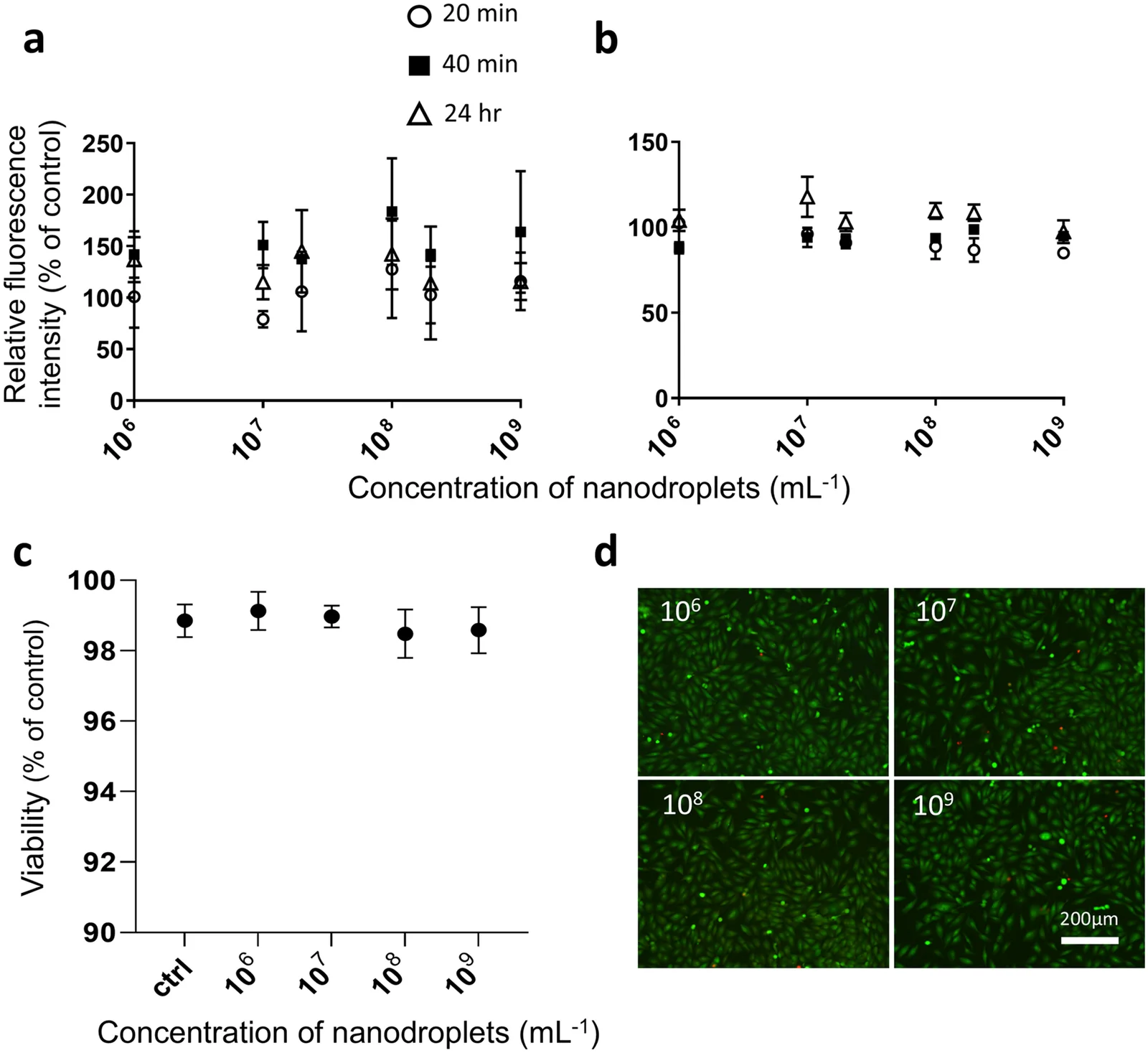
NDs have no significant effect on MG63 or BMSC metabolism. There were no significant effects of any concentration of NDs on MG63 metabolism, as measured by alamarBlue™ fluorescence normalised to vehicle control (100%), except between 40 min and 20 min for 1 × 10^7^ mL^−1^ (*p* = 0.013) (a). Similarly, there was no concentration-dependent effect on BMSC metabolism (b). In addition, there was no effect of NDs at any concentration on cell viability, measured using a LIVE/DEAD assay (c) with representative images shown in (d) (green = live; red = dead). Data points are mean values of 3 separate measurements, with error bars representing the corresponding standard deviation.

To test the biocompatibility of NDs with bone-derived cells, we incubated either MG63 cells or primary HBMSCs with NDs for up to 24 hours and measured cell metabolism, a proxy for cell viability, using alamarBlue™. For MG63 cells (Fig. 4a), there was no concentration-dependent effect of NDs at any timepoint, although there were small increases in relative fluorescence intensity at 40 minutes post-ND addition compared to 20 minutes (significantly only at a concentration of 1 × 10^8^ mL^−1^; *p* = 0.013, *n* = 3). At 24 hours however, the fluorescence intensity declined to levels similar to those at 20 minutes. For BMSCs (Fig. 4b), there was a minor decrease in cell metabolism with respect to ND concentration at the initial timepoint (20 minutes; *p* = 0.012), but not at other timepoints. However, an intermediate concentration of NDs promoted cell metabolism relative to control at 24 hours (1 × 10^7^ mL^−1^; *p* = 0.01, *n* = 3). At all ND concentrations tested there were time-dependent significant increases in cell metabolism, likely reflecting cell growth (*p* < 0.01 for all concentrations). In addition, there were no direct effects on cell viability, as measured using a LIVE/DEAD assay 24 hours following addition of NDs (Fig. 4c and d). These data indicate that NDs can be used without having an adverse effect on cell metabolism.

### BIO released by ultrasound stimulation of NDs is active, and induces Wnt signalling in reporter cells

**Fig. 5:**
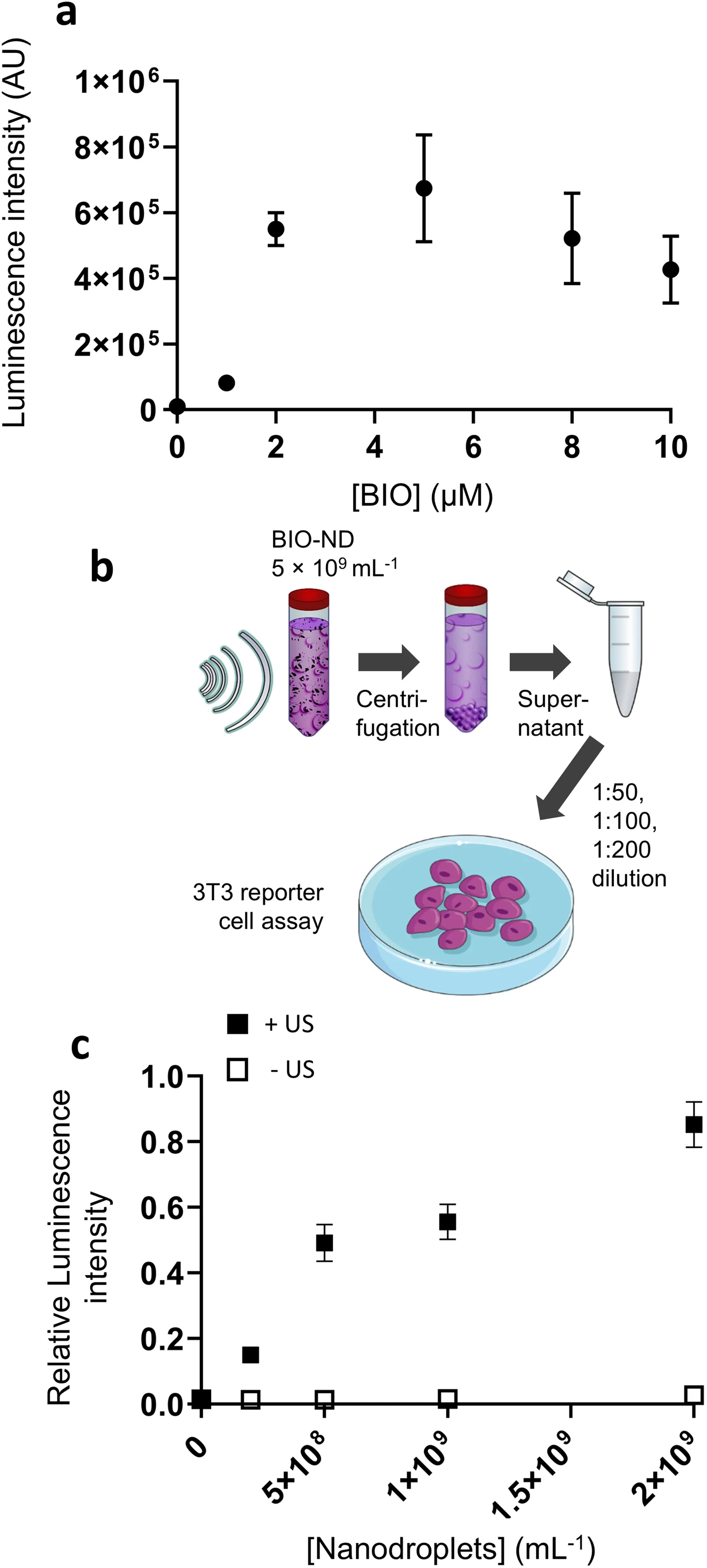
BIO, loaded in NDs and released with ultrasound, retains signalling activity in reporter cells. BIO induces a concentration-dependent biphasic induction of Wnt signalling in reporter cells with a maximum at ∼5 µM (a). BIO-NDs were exposed to ultrasound, before the supernatant was collected by centrifugation and applied to 3T3 reporter cells at dilutions equivalent to ND concentrations in the original preparation (b). BIO, released in this manner from NDs caused a dose-dependent increase in Wnt signalling to a maximum of a factor of 0.85 relative to free BIO (c). Data points are mean values of 3 separate measurements, with error bars representing the corresponding standard deviation.

We next tested the hypothesis that ultrasound-activated BIO release can be used to induce Wnt signalling. For these studies, a reporter cell line was used, which expresses luciferase under the control of a Wnt-responsive promoter (TCF/LEF). The magnitude of Wnt activation can therefore be determined by luminescence resulting from the luciferase-catalysed oxidation of d-luciferin, which is proportional to luciferase production. As expected, BIO induced a dose-dependent increase in luminescence from 0 to 5 µM with a decline thereafter (Fig. 5a), which is likely due to the toxicity of this compound at higher concentrations.^26^ To determine whether encapsulation and release inhibited the activity of BIO, we next exposed BIO-NDs to ultrasound as above and collected the supernatant following centrifugation. This supernatant was then added to cell culture medium at defined volumes that were equivalent to ND concentrations in the original sample (Fig. 5b). The supernatant caused a dose-dependent increase in Wnt-signalling activity with respect to ND concentration, with a maximum at a value of 85.2% relative to the activity of free BIO at 5 µM (Fig. 5c). These data indicate that active BIO can be released by NDs under ultrasound exposure and stimulates Wnt signalling in a cell line, indicating that encapsulation in NDs and ultrasound exposure do not affect BIO stability.

### BIO released by ultrasound stimulation of NDs in the presence of cells induces Wnt signalling

**Fig. 6:**
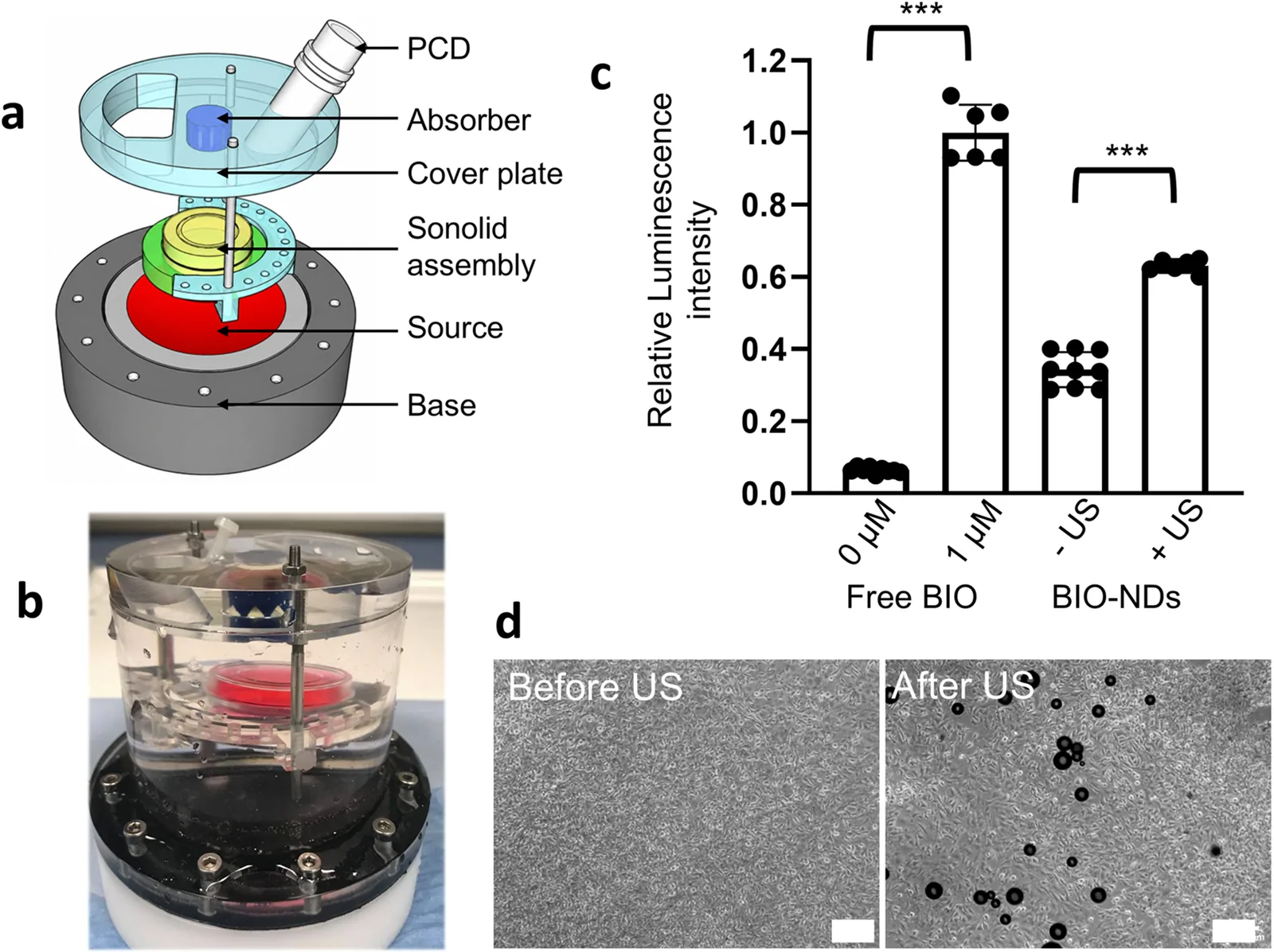
Activation of BIO-NDs in the presence of cells induces Wnt signalling activity. The SAT-D device, as shown in the schematic in (a) and set-up with a cell culture in (b), was used to stimulate cells in the presence of NDs. Following US stimulation, there was a significant increase in Wnt signalling activity relative to unstimulated controls, although BIO-NDs in unstimulated samples also had significant activity relative to controls (c), *** denotes *p* < 0.001. US induced a visible vaporisation of NDs (d); scale bar indicates 200 µm.

To determine whether ultrasound can be used to induce release of BIO from NDs and subsequent signalling directly in the presence of cells, we next used the SAT2 device^24^ allowing for ultrasound stimulation in cell cultures (Fig. 6a and b). 3T3 cells, plated in ibidi® dishes containing either BIO-NDs at a concentration of 1 × 10^8^ mL^−1^ (corresponding to a concentration of 1 µM BIO) or vehicle controls in growth medium, were stimulated in the SAT2 device with ultrasound at a peak negative pressure of 0.75 MPa, frequency of 0.9 MHz, pulse repetition frequency of 1000 Hz and 10% duty cycle, for 30 seconds. Higher acoustic pressures were also tested but as significant cell detachment occurred at pressures > 1 MPa, it was not possible to measure the effect of BIO release at these greater pressures. Cells were then incubated overnight before measurement of Wnt signalling activity, relative to free BIO, at a concentration of 1 µM.

Even in the absence of ultrasound, BIO-NDs induced signalling activity in 3T3 cells, to a value of 34.3 ± 6% of the 1 µM positive control (Fig. 6c). However, ultrasound stimulation resulted in an activity of 63.1 ± 1.9% of this positive control, which was comparatively 83.9 ± 5.5% greater than the BIO-ND sample that was not stimulated with ultrasound (*n* = 9; *p* < 0.001). In US-stimulated medium supernatant (after centrifugation and removal of remaining NDs) there was a final concentration of 0.25 ± 0.06 µM BIO measured by HPLC, indicating a release of approximately 25%. Empty NDs induced no increase in Wnt signalling regardless of the presence of US, and NDs did not inhibit Wnt signalling in the presence of free BIO, indicating that NDs or their constituents do not interfere with BIO-induced Wnt signalling.

Prior to ultrasound stimulation, there was no evidence of gas microbubbles illustrative of spontaneous ND vaporisation; however, on stimulation with ultrasound, there was evidence of immediate microbubble formation in the device by macroscopic observation as well as by microscopy images of 3T3 cultures post-stimulation (Fig. 6D) that was not present in the absence of ultrasound stimulation. Despite indications of ND vaporisation in microscopy images, the spectral content of the PCD recordings indicated no significant increase in broadband signal in ND samples relative to medium samples (∼83 dB for both; SI Fig. S1). However, in degassed PBS the ND formulations were both observed to vaporise and an increase in broadband signal was recorded compared to ND-free controls (SI Fig. S2), indicating that it is likely that in complex growth medium acoustic emissions from endogenous microbubbles may mask broadband signal from cavitating NDs. In summary, these results indicate that ultrasound can be used to induce BIO release from NDs co-incubated with cells.

### NDs passively release BIO in growth medium

**Fig. 7:**
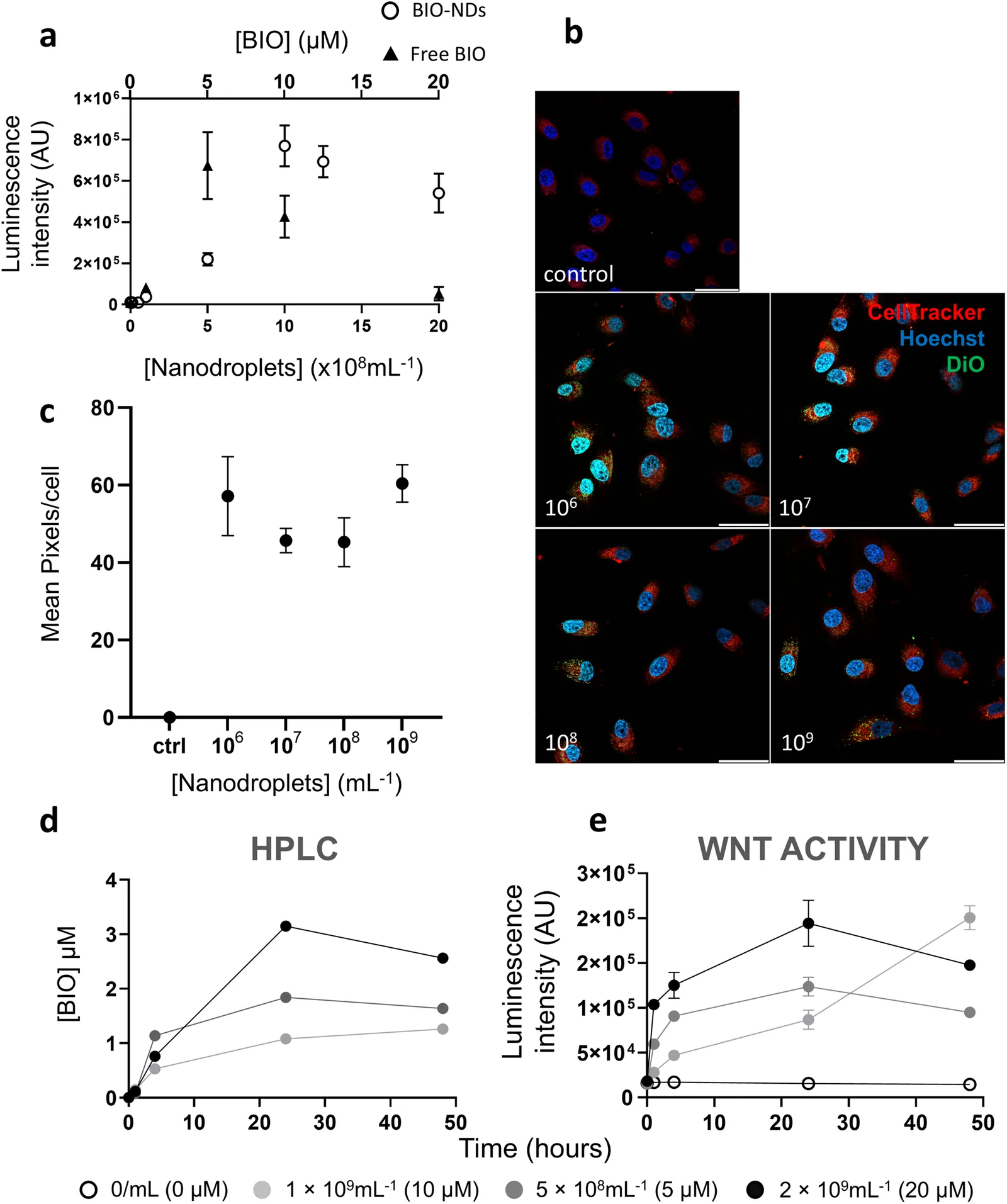
NDs activate Wnt signalling in the absence of ultrasound by both cell uptake and unstimulated BIO release. Addition of BIO-NDs to 3T3 cells activates Wnt signalling in a dose-dependent manner (a). DiO-labelled NDs are taken up by 3T3 cells (b and c). BIO is passively released from NDs, as reflected by an increase in the concentration of BIO in serum-containing medium with respect to time measured by HPLC (d) or in Wnt signalling activity in reporter cells (e). Data points are mean values of 6 separate measurements, with error bars representing the corresponding standard deviation.

The observation of BIO activity in cultured cells exposed to BIO-NDs in the absence of ultrasound suggests there was ND uptake by cells, passive release of BIO into the medium, or ND instability and non-US specific vaporisation and release of BIO during prolonged culture. Supporting the former point, in previous work, we have shown that labelled NDs are readily internalised by cancer cells, suggesting that intracellularly delivered BIO may contribute to signalling.^28^ To assess whether NDs could intrinsically activate Wnt signalling, we first cultured 3T3 cells in the presence of increasing concentrations of BIO-NDs compared to free BIO. BIO-NDs elicited a maximal Wnt signalling activity at a concentration of 1 × 10^9^ mL^−1^ (equivalent to a bulk BIO concentration of 10 µM) (Fig. 7a). There was a decline in concentration thereafter, likely related to cell toxicity as BIO is known to be toxic at sufficiently high concentrations.^28^ Concurrently, it was evidenced by labelling NDs with a lipophilic fluorophore that NDs were internalised by 3T3 cells (Fig. 7b and c).

In aqueous buffer, NDs did not release BIO except in the presence of ultrasound stimulation (Fig. 3c). To test ND stability in serum-containing medium, BIO-NDs were stored in growth medium for up to 48 hours, before centrifugation and collection of the supernatant. The supernatant was then added to 3T3 reporter cells at concentrations equivalent to 5 × 10^8^, 1 × 10^9^ and 2 × 10^9^ NDs mL^−1^ (equivalent to bulk BIO concentrations of 5, 10 and 20 µM) and analysed by HPLC. The results showed a time-dependent release of BIO for all ND concentrations (Fig. 7d) with ∼15–20% release at 24 h, reflected in the increased Wnt signalling activity of cells at this time (Fig. 7e). The decline at 48 hours is likely due to some lability of the BIO molecule, as we found that BIO stored for 48 hours in growth medium was significantly less active than that stored for 24 hours.

## Discussion

In this study, we developed PFB-NDs that could encapsulate a small molecule of the Wnt signalling pathway, BIO, when stored in aqueous buffer. Under ultrasound stimulation, the NDs could be vaporised to release their payload on demand. Although PFB-NDs were not stable over periods of hours in serum-containing medium, they had no detrimental effect on cell metabolism and could be induced to release BIO by ultrasound exposure, activating Wnt signalling.

In previous work, we found that a hydrophobic fluorophore, Nile red, can be incorporated into NDs and released on ultrasound exposure.^28^ Nile red is a non-polar molecule with a predicted log *P* of 3.8, similar to that of BIO, with an estimated log *P* of 3.9.^26^ We did not explore the mechanism by which either molecule interacts with NDs, but we consider it likely that non-polar molecules may be sequestered in the phospholipid membrane of NDs. Perfluorocarbon molecules such as PFB are inherently both lipophobic and hydrophobic, and partition from both aqueous and non-polar solvents. This makes dissolution of BIO or Nile red in the PFC phase unlikely. It is well known that liposomal carriers, with similar lipid chemistries to the NDs we fabricated, can incorporate hydrophobic small molecules (*e.g.* doxorubicin in the commercial preparation Doxil®)^29^ or proteins (*e.g.* Wnt3A),^30,31^ and that interaction between small molecules and lipids can occur in lipid monolayers as well as lipid bilayers.^32^ In addition, we have also found that BIO is stably incorporated in polymeric membranes composed of PCL-PEG^26^ similarly to hydrophobic molecules such as DiD.^33^ Combined with the small increase in size we observed by DLS and NTA for BIO-loaded *vs.* control PFB-NDs, we hypothesise that BIO is incorporated in the lipid monolayer of these particles. Further measurements, such as NMR, will be necessary to investigate this possiblity, however.

Formulations of BIO-loaded PFB-NDs could be induced to release their cargo in medium buffer on exposure to 1 MHz ultrasound at a peak negative pressure of 1.1 MPa, with 5000 cycle pulses and PRF of 100 Hz, as detected using HPLC (Fig. 3c). We found that the peak negative pressure required for release was lower than that identified as the ND vaporisation threshold in our previous work.^34^ This is potentially because the earlier study was performed in PBS rather than serum and under flow conditions at a lower PRF. In the present study ∼5 min exposure to ultrasound induced > 80% drug release. The BIO that was released from NDs after ultrasound stimulation was confirmed to be active. Supernatants taken from US-treated BIO-ND samples induced activation of Wnt signalling in 3T3 cells, with a maximum activity at 85% of free BIO using supernatant from NDs at the maximum concentration (Fig. 5c). This is consistent with previous work demonstrating drug activity after ultrasound stimulation from a variety of ND chemistries.^4,15,35^ The stability of NDs in aqueous buffer was also supported by the lack of any appreciable activity or chemical detection of BIO in supernatants unstimulated with ultrasound.

In experiments using the SAT2 device, which allows BIO-ND activation in the presence of cells, we initially found that ultrasound pressures greater than 1 MPa led to detachment of cells, making it impossible to make measurements of activity at the pressures used to promote drug release *in vitro.*^28^ However, it was subsequently found that a lower peak negative pressure of 0.75 MPa was sufficient to produce a significantly higher induction of Wnt signalling activity in cells compared to sham controls. PCD measurements did not reveal a significant increase in broadband signal relative to ND-free controls (SI Fig. S1). It is important to note, however, that these experiments were carried out in serum-containing growth medium that was not degassed prior to use (oxygenation of the growth medium is necessary for cell survival) and we noted the presence of large bubbles in US-treated samples compared to sham treated controls. We therefore consider it possible that pre-existing microbubbles in the medium may lead to a high cavitation noise level that masks additional emissions from ND vaporisation. It is also possible that the acoustic activity of these microbubbles and/or the presence of serum promotes ND vaporisation at lower acoustic pressure amplitudes.^36^ Further work is needed to investigate these effects.

In the SAT2 experiments where ultrasound stimulation was conducted in the presence of cells and NDs, it was clear that sham control samples that were not exposed to ultrasound also had significant Wnt-signalling activity, albeit at a level ∼55% of the US-treated control (Fig. 6). We considered this likely to have been due to a combination of both uptake of NDs and leakage of BIO from NDs in serum-containing medium. There is some evidence in the literature suggesting that NDs may be internalised by cells^28,37–39^ and this is consistent with the dose-dependence of Wnt signalling that we observed when NDs were incubated with 3T3 cells. In addition, when fluorescently labelled NDs were incubated with 3T3 cells, subsequent microscopy indicated intracellular uptake of fluorescent material; although unfortunately the spatial resolution of the microscope was insufficient to confirm whether or not these were intact ND (Fig. 7b). Interestingly, however, the peak signalling activity for BIO added directly to the growth medium was at 5 µM as opposed to 1 × 10^8^ mL^−1^ BIO-NDs (which added at this concentration and, if evenly distributed in the growth medium, would be equivalent to a concentration of 10 µM). This indicates that some BIO remained unavailable to cells. In addition, over the period of the Wnt signalling assay (18 hours), a significant amount of BIO leaked out of BIO-NDs, with a maximum of ∼15% release measured at 24 hours by HPLC (and a value of ∼30% activity detected by Wnt signalling (Fig. 7d & e)). This suggests that NDs have lower stability in serum-containing medium compared to aqueous buffer. This is again consistent with previous studies^40^ and likely due to nanodroplet coalescence promoted by serum proteins combined with the relatively low boiling point of PFB. It is also consistent with the reduction in ultrasound pressure required for ND activation.

Our study was designed to determine the *in vitro* stability and triggered release of active BIO from NDs. Further pre-clinical testing and the ultimate application of this technology will require a fuller understanding of ND stability and drug release in the dynamic *in vivo* environment. At present the development of the ND formulation described in our study is limited by the lack of stability in serum-containing medium. In future, however, stability may be improved by a variety of means, including increase steric stabilisation at the droplet interface by increased PEGylation,^41,42^ replacement of phospholipid surfactants with polymer interfaces that prevent protein adsorption^43^ or the use of PFCs with a higher boiling point, for example PFP. In bone repair applications, NDs may be delivered as a component of a scaffold or carrier implanted or injected locally; or as circulating agents injected intravenously. It is notable that PFC NDs have previously been used to modulate the structural properties of biomaterial scaffolds, commonly used in tissue engineering^44,45^ and have been used for gene delivery in applications in bone repair.^21^ We anticipate that agonists of Wnt signalling, such as BIO, might be used in similar way for controlled release to stimulate bone healing. There are now many studies, reviewed recently by Wang *et al.*,^46^ indicating positive results in the delivery of drugs using US-activated NDs in other disease contexts. For example, ND mediated delivery of doxorubicin has been shown to have improved efficacy in treating a model of prostate cancer compared with the drug alone.^47^ This result was at least in part attributable to PFC's capacity for dissolving oxygen to enable tumour sensitisation. Since oxygen is also a key regulator of bone repair^48^ and systemically-delivered nano-sized particles accumulate at fracture injury sites,^49^ similar co-delivery strategies might be adopted for bone repair. Notably, larger microbubble ultrasound contrast agents overtly transit bone fractures *in vivo*, as demonstrated by imaging studies, indicating that poorly-healing bone fractures can be highly perfused with vasculature and are available for ultrasound-based targeting.^50^ Moreover, the ultrasound exposure parameters used in our study (*i.e.* ∼1 MHz centre frequency, ∼1 MPa peak negative pressure, 5000 cycles and 100 Hz PRF) are well within the range currently used for ultrasound physiotherapy, including for fracture healing, which will be beneficial for clinical translation.

## Conclusions

In summary, we have developed NDs containing a small molecule that stimulates Wnt signalling, BIO. The NDs can be stored stably in buffer and can be stimulated to release their payload under ultrasound stimulation, resulting in activation of Wnt signalling in a reporter cell line. However, NDs were less stable in serum-containing medium over time periods of hours. Future work should focus on improving ND stability in serum and on investigating further the mechanisms governing ND vaporisation in physiological media. Moreover, ND activity could be assessed using a broader range of cell types that are representative of distinct tissue types and/or of different functional structures of bone.

## Author contributions

Conceptualization: NDE, DC, ES; methodology: QW, NDE, DC, ES; investigation: AP, QW, JM, SF; visualization: AP, QW; supervision: NDE, DC, ES; funding acquisition: NDE, DC, ES; project administration: NDE, DC, ES; writing – original draft: AP, NDE, QW; writing – review & editing: NDE, DC, ES.

## Conflicts of interest

There are no conflicts to declare.

## Supplementary Material

PM-003-D5PM00186B-s001

## Data Availability

The data supporting this article have been included as part of the supplementary information (SI) Lab books and other physical information are stored for 7 years at the University of Southampton or the University of Oxford. Supplementary information (SI), which comprises SI Fig.1 and SI Fig. 2, showing the spectral content of PCD recordings in ND-containing medium samples or in degassed PBS samples, is available. See DOI: https://doi.org/10.1039/d5pm00186b.
