# Supplementary material for: Ultrasound-triggered release of an active Wnt signalling agent (6-bromoindirubin-3-oxime) from perfluorocarbon nanodroplets
Source: RSC Pharm. 2026 Jul 13;3(5):1253–65. doi: 10.1039/d5pm00186b (PMC13544251; doi:10.1039/d5pm00186b)
Supplement: Supplementary file 1 [file PM-003-D5PM00186B-s001.pdf]

This journal is © The Royal Society of Chemistry 2026

Supplementary Figure 1

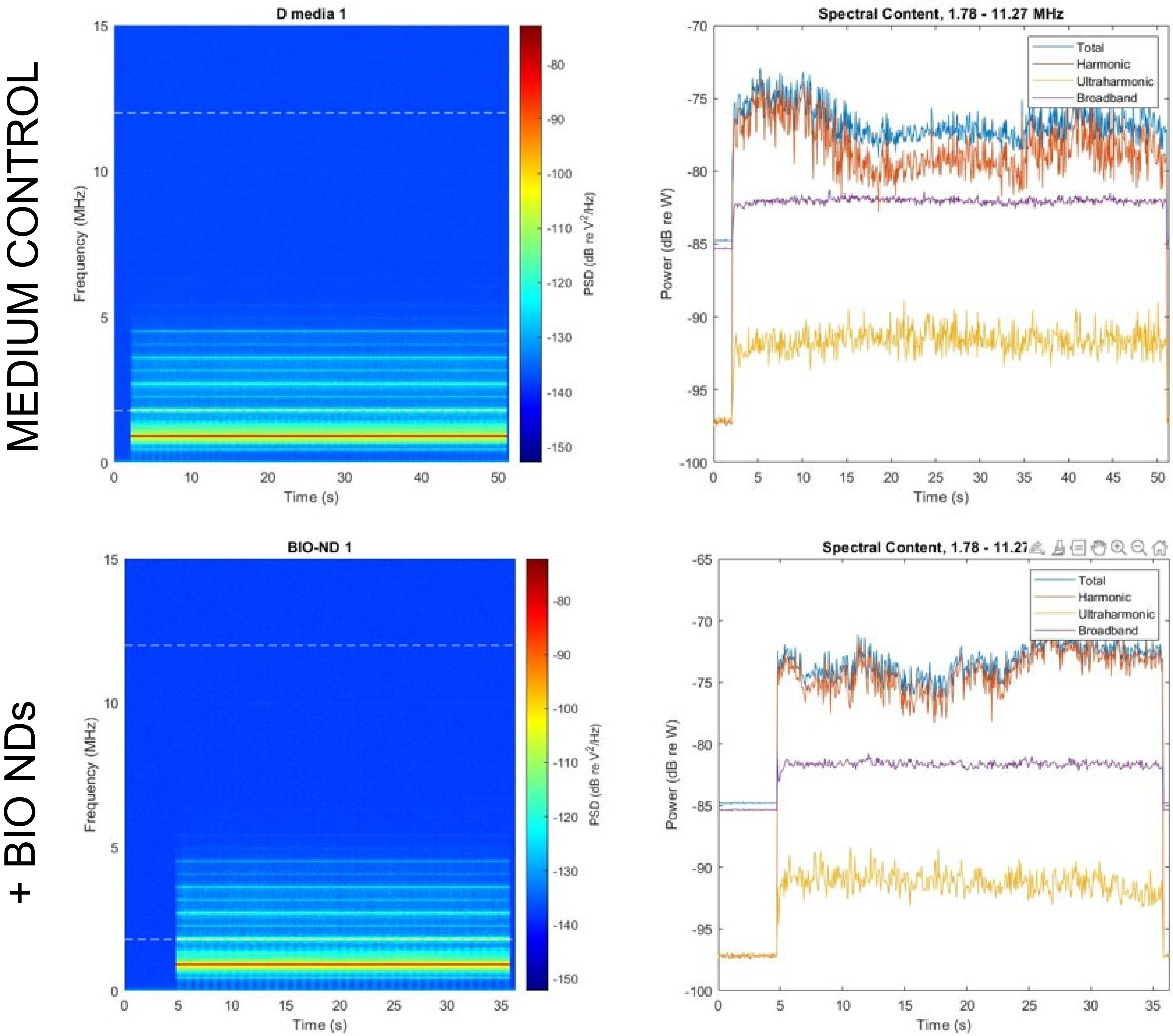

Supplementary Figure 1. In cell culture medium, there was no detectable difference in power with (lower) or without NDs (upper).

Supplementary Figure 2

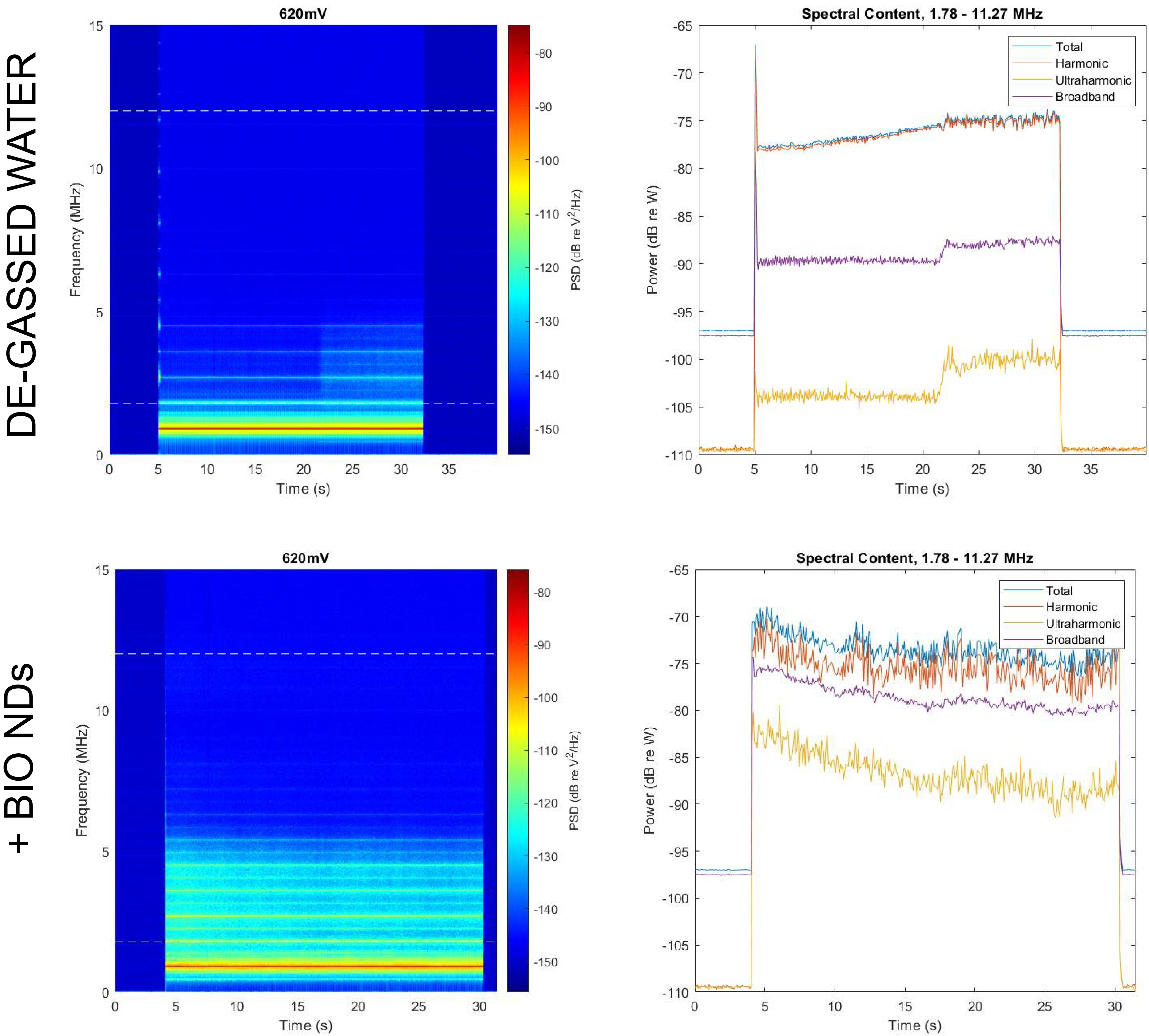

Supplementary Figure 2. In degassed water, there was an increase in power in ND-containing samples (lower) compared to those without (upper).
